# Health Protective Behavior in Occupational Health Practice: A Concept Analysis

**DOI:** 10.1002/hsr2.71020

**Published:** 2025-07-09

**Authors:** Fenggang Liu, Juanjuan Wang, Weeraporn Suthakorn, Li Liao

**Affiliations:** ^1^ International PhD Degree Program, Faculty of Nursing Chiang Mai University Chiang Mai Thailand; ^2^ Hengyang Medical School, Cardiac Interventional Imaging Center, The First Affiliated Hospital University of South China Hengyang City Hunan Province People's Republic of China; ^3^ School of Nursing University of South China Hengyang City Hunan Province People's Republic of China; ^4^ Occupational Health Department Hengyang Traditional Chinese Medicine Hospital Hengyang City Hunan Province People's Republic of China

**Keywords:** analysis, conceptualization, health‐related behavior, nursing, occupational health

## Abstract

**Background and Aims:**

Health protective behavior (HPB) is critical in reducing occupational accidents and diseases, yet prior research lacks conceptual clarity. Existing definitions vary widely, ranging from compliance with medical advice to broader hazard avoidance, and fail to holistically address occupational contexts. These inconsistencies hinder the development of targeted interventions and standardized practices in workplace health protection. This study aims to analyze and redefine HPB within occupational health practice to address these gaps.

**Methods:**

Using Walker and Avant's concept analysis framework, a systematic literature review was conducted across PubMed, SCOPUS, CINAHL, and other databases. After screening 1370 records, 19 articles met the inclusion criteria. Data were analyzed to identify defining attributes, antecedents, consequences, and empirical referents.

**Results:**

Five key attributes characterize HPB: (1) proactive assessment and control of environmental hazards, (2) compliance with safety guidelines, (3) concern for the social environment, (4) personal health practices, and (5) health maintenance. Ten antecedents were identified, including hazard awareness, access to resources, self‐efficacy, workplace safety climate, etc. Six consequences emerged, such as enhanced well‐being, reduced occupational risks, cost savings, and improved quality of life, etc. Model, borderline, and contrary cases further clarified the concept. HPB was redefined as majorly focused on behaviors integrating hazard control, safety adherence, social engagement, and health preservation.

**Conclusion:**

This analysis provides a unified conceptualization of HPB, addressing prior ambiguities. The findings offer a foundation for developing interventions, instruments, and policies to strengthen occupational health practices. Future research should explore contextual factors influencing HPB and test targeted strategies to promote worker safety and well‐being.

## 1 Introduction

Occupational accidents and work‐related diseases remain critical global challenges [1]. Annually, ~2.3 million workers die due to occupational hazards, a figure surpassing fatalities from tuberculosis, AIDS, and road accidents combined [2, 3]. Nonfatal occupational injuries affect over 300 million workers, leading to disabilities, absenteeism, and economic burdens [4]. Common risks include exposure to chemical agents, physical hazards (e.g., radiation, noise), ergonomic strains, and psychosocial stressors [5]. These risks not only threaten individual health but also strain healthcare systems, reduce productivity, and destabilize communities [6].

To mitigate these risks and protect workers, the ongoing prevention of accidents and work‐related diseases using a protective approach assumes a crucial role [7, 8]. This requires a concerted effort from various stakeholders, including employers, employees, regulatory bodies, and occupational health professionals [9]. Health protective behavior (HPB) has been identified as one of the protective approaches taken by the workers to protect their health at various work related and regular life occasion [10, 11, 12]. Pender defined health promotion as activities directed toward increasing the level of well‐being and self‐actualization, whereas health protection was defined as activities directed toward decreasing the probability of experiencing health problems by active protection against pathologic stressors or detection of health problems in the asymptomatic stage [13, 14]. Health protection refers to a set of strategies or systems put in place to safeguard oneself when preventive measures prove inadequate. Therefore, unlike generalized health promotion, HPB specifically addresses occupational hazards through proactive measures such as adherence to safety protocols, hazard avoidance, and health maintenance. For instance, radiation poses a hazard to personnel working in radiological departments; from a prevention standpoint, our goal is to eliminate radiation exposure [15]. However, radiation is also required as a treatment method for patients, so our focus shifts to protecting healthcare personnel by minimizing their exposure dose through complying with equipment safety guidelines exemplified by HPB in practice. Effective HPB reduces injury rates, enhances job satisfaction, and fosters safer work environments [16, 17].

Despite its importance, HPB lacks conceptual clarity. Early definitions, such as Harris and Guten's broad characterization of HPB as “any behavior performed to protect health” [18], contrast sharply with narrower interpretations. Berkanovic [19] framed HPB as compliance with medical advice, while Vinehout [20] focused solely on disease prevention, Naje and Mahdi [21] focused on dietary habits, physical activity, and health follow‐up, and Aker and Aiken [22] aimed to protect health and minimize the risk of infection such as handwashing, wearing a facemask, and disinfecting surfaces. These inconsistencies create ambiguity in occupational contexts. For example, definitions emphasizing medical compliance overlook workplace‐specific risks like chemical exposure, while those focused on disease prevention neglect preventive actions like ergonomic adjustments [5, 23]. Furthermore, existing frameworks rarely integrate psychosocial factors (e.g., workplace culture) or address the interplay between individual and organizational responsibilities [24].

The absence of a unified HPB conceptualization hinders the development of targeted interventions, standardized training programs, and evidence‐based policies. For instance, conflicting definitions complicate the design of workplace safety audits or employee health assessments. This study addresses these gaps by systematically analyzing HPB attributes, antecedents, and consequences within occupational settings. Proposing a redefined HPB framework tailored to workplace contexts and providing actionable insights for policymakers and occupational health practitioners to enhance hazard mitigation strategies. By clarifying HPB in occupational health practice, this study aims to strengthen occupational health policies, improve compliance monitoring, and foster safer, more resilient workplaces.

## 2 Methods

### 2.1 Concept Analysis Method

Walker and Avant's concept analysis approach has been frequently used in multiple disciplines due to its structured, systematic process for defining ambiguous concepts. Unlike Rodgers' emphasis on concept evolution across contexts, it ensures rigor in distinguishing HPB from related constructs like health promotion or disease prevention. Therefore, it was selected as the concept analysis method. A systematic approach including eight steps was used: “(1) Select a concept; (2) Determine the aims or purposes of analysis; (3) Identify all uses of the concept that can be discovered; (4) Determine the defining attributes; (5) Identify a model case; (6) Identify borderline, related, or contrary cases; (7) Identify antecedents and consequences; and (8) Define empirical referents” [25]. Ethical approval of the study was granted from the First Affiliated Hospital of University of South China, No: 2024LL0722001.

### 2.2 Inclusion and Exclusion Criteria

Inclusion criteria were set as follows. Works were required to be published in English, and the title of any discipline's article associated with HPB related to occupation was included in the search. As HPB is a concept associated with humans, literature related to humans was included in the process. Availability of full texts could help us to explore the research details; hence, “full text” as a filter was used in the searching process. The references cited in every article were reviewed carefully for notable consistencies among them. Bibliographies and other sources were pulled in addition to the formal search strategy (unpublished literature, books, and nonpeer‐reviewed articles, etc.). In our initial search, we found that there were not many articles in this area, so we did not limit the time of publication. The authors focused on the definitions and description of health protection, and reviewed all the literature, versions of dictionaries, book chapters, and web pages. All the material searched was pulled into endnotes to perform the analysis.

Exclusion criteria were articles that were duplicates, articles in which the keyword was used only as a predicate or was anonymous, and irrelevant articles.

### 2.3 Data Sources

Wide databases were selected as data sources, such as PubMed, SCOPUS, Cumulative Index to Nursing and Allied Health Literature (CINAHL), Academic Search Ultimate, ProQuest, Engineering Source, and Education Source. Keywords, “health protection” “protective behavior” “health behavior” “occupational protect*” “protection behavior” were respectively connected with “work*” or “occupation” or “career” or “labor” or “job” by using Boolean rule “AND,” were set to do the search with the filter of “title/abstract,” and the inclusion and exclusion criteria were formulated. In SCOPUS, we used database‐specific filters and set the search string (TITLE‐ABS‐KEY(“health protective behavior” AND (occupation OR work* OR job)))*, while in PubMed, we used filters “title/abstract” to ensure relevance. In addition, other databases were integrated into the Chiang Mai University database, which were searched by the same method as in PubMed.

**Figure 1:**
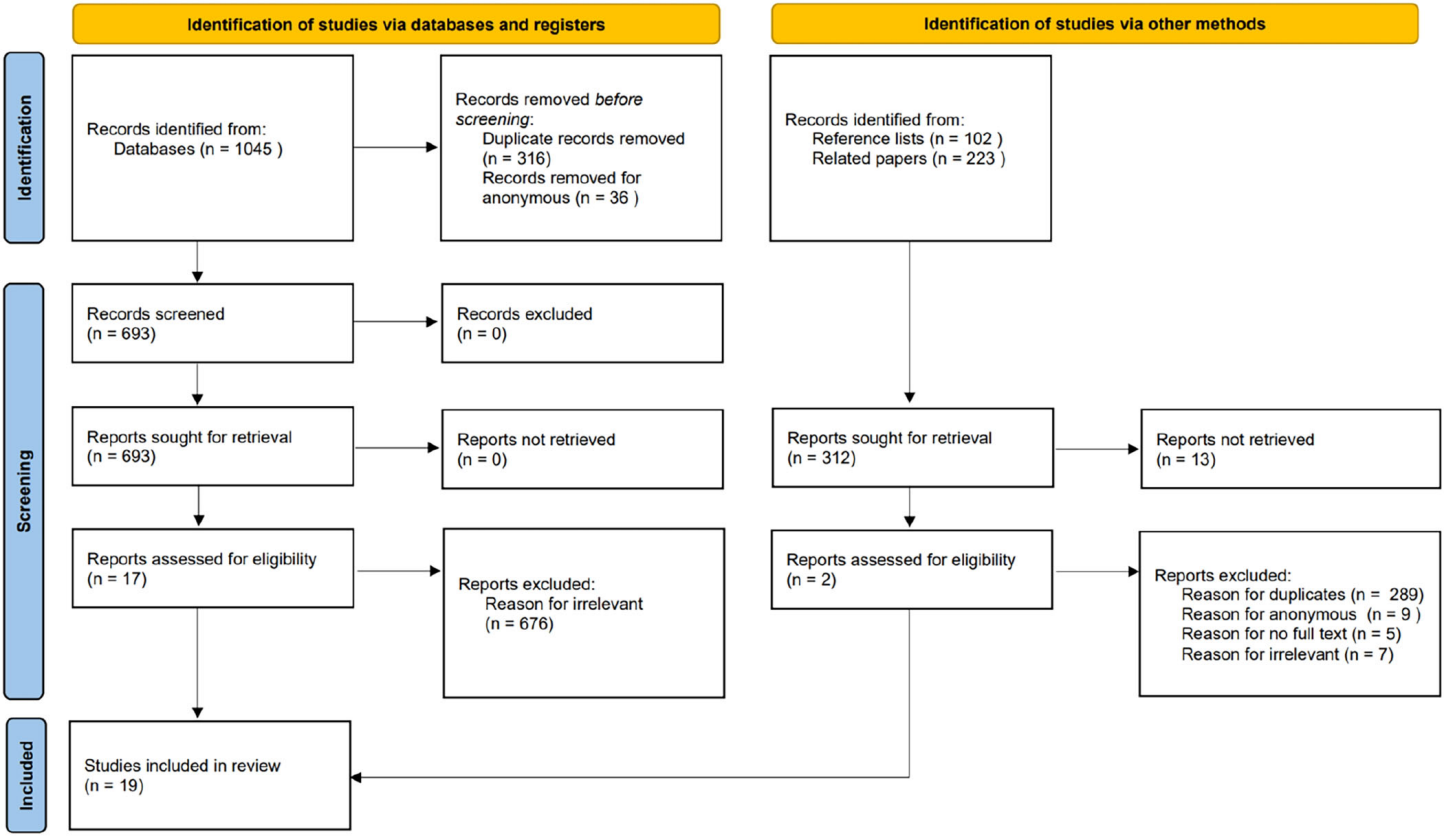
Flowchart of the study selection process.

Under the inclusion criteria, 1370 articles were found. After utilizing the duplicates tool in Endnotes, 605 were screened out as duplicates, and 45 were dropped as anonymous. The authors reviewed the titles and abstracts of all the remaining articles (*n* = 720), and 701 papers were excluded for irrelevance. After reexamining the abstracts of the remaining articles, papers which could explain at least one of the attributes, antecedents, consequences, factors, or measurement methods of health protection were used as the final list by Microsoft Office Excel software (*n* = 19), as shown in Figure 1.

## 3 Results

### 3.1 Uses of the Concept

From the literature review, the authors found that health protection is a commonly used concept in multiple disciplines, including sociology, psychology, nursing, public health, management, and education. It can be used in terms of activities [26], actions [27], processes [28], behaviors [14, 29], or interventions [30] to protect humans against hazards in the nursing discipline. Health protection used as behavior decreased the probability of becoming ill in psychology discipline [31]. In public health, it can be used as health protective functional elements [32], measures [33, 34], activities [35], actions [34, 36], or processes [37] to safeguard human health, or as discipline to protect the public and limit hazards [38]. It can be used as a procedure to protect humans in education [39] and measures [40], or factors to prevent hazards and promote health in sociology [41], and procedures [42] or activities for positive health [43] in the management discipline.

### 3.2 Defining Attributes

**Figure 2:**
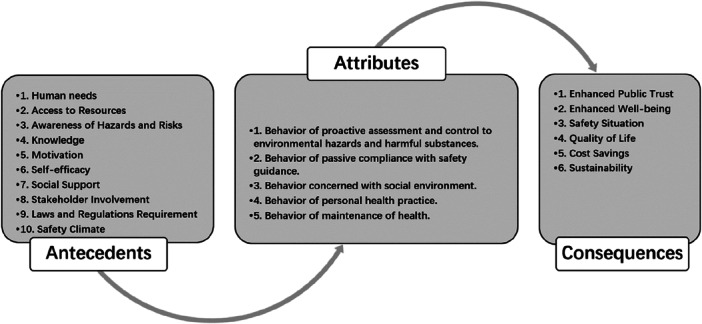
Health protective behavior in occupational health practice: antecedents, attributes, and consequences.

From the analysis, we found that the scope of health protection is widely used. Its attributes can be divided into abstract and concrete aspects. The details are listed with the authors, years, disciplines, definitions, antecedents, attributes, and consequences in Table 1. The key characteristics of health protection were listed as attributes, the necessary events for health protection were listed as antecedents, and the results of health protection were listed as consequences. As in a psychology dictionary, behavior was defined as “an action, activity, or process which can be observed and measured” [44]. Therefore, the attributes were reclassified into several brief phrases, as shown in Figure 2.

**Table 1:** Overview of selected references.

Disciplines	Definition	Attributes	Antecedents	Consequences
*Nursing (n = 6)*				
[26]	Health protection was defined as activities “directed toward decreasing the probability of experiencing health problems by active protection against pathologic stressors or detection of health problems in the asymptomatic stage.”	1. Activities of actively protecting or detecting health‐related factor	1. Awareness 2. Knowledge 3. Motivation 4. Access to resources	1. Decreasing the probability of experiencing health problems 2. Health outcomes 3. Risk reduction
[27]	Health protection was defined as actions in protecting people from hazards which damage their health. With regard to the scope of health protection, there was a consensus from the consultation to include infections and diseases due to exposure to nonbiological hazards, but not injuries.	1. Actions of environmental hazard avoidance 2. Actions of harmful substance avoidance	1. Perception of hazards 2. Knowledge 3. Awareness 4. Resources and access 5. Self‐efficacy	1. Reduced exposure 2. Improved well‐being 3. Quality of life 4. Occupational and environmental safety
[28]	Health protection refers to disease and injury prevention and the promotion of positive health via legislation, policy, regulation, and codes of conduct.	1. Actions of prevention and promotion by compliance with safety guidance 2. Concern with social environment	1. Perceived threat of disease and injury 2. Motivation 3. Attitudes 4. Personal values and beliefs	1. Positive social norms 2. Enhanced public trust 3. Personal and organizational safety
[14]	Health protection is defined as the “behavior motivated by a desire to actively avoid illness, detect it early, or maintain functioning within the constraints of illness.”	1. Active behavior	1. Awareness of health risk 2. Physical and mental abilities	1. Personal achievement and self‐fulfillment
[30]	Health protection refers to interventions that help reduce health risks by modifying the environment to support healthier living.	1. Interventions by modifying the environment	1. Factors identification 2. Knowledge 3. Stakeholder involvement	1. Healthier living 2. Sustainability
[29]	Health protection refers to behaviors that decrease one's probability of becoming ill.	1. Behavior decreased probability of becoming ill 2. Personal health practice	1. Social support 2. Health knowledge 3. Beliefs and attitudes 4. Self‐efficacy	1. Quality of life 2. Longevity
*Psychology (n = 1)*				
[31]	Health protection refers to behaviors that decrease one's probability of becoming ill.	1. Behavior decreased probability of becoming ill	1. Perceived susceptibility and severity 2. Knowledge 3. Motivation 4. Safety climate	1. Reduced illness incidence 2. Enhanced public trust 3. Cost savings
*Public health (n = 7)*				
[32]	Health protection is defined as those elements of the public health function which protect the population against communicable diseases and noncommunicable environmental hazards (such as chemical fires and radiation threats).	1. Actions of environmental hazard avoidance 2. Actions of harmful substance avoidance	1. Perception of hazards 2. Awareness of risk 3. Knowledge of identification	1. Occupational health 2. Positive social influence
[33]	Health protection refers to measures adopted to safeguard the health of the community as a whole: clean water, good sanitation, safe roads and playgrounds, and sound policies in home‐building.	1. Measures of harmful substance avoidance to safeguard health	1. Potential health risk 2. Laws and regulations requirement	1. Safety situation 2. Enhanced public trust
[38]	Health protection is an action that seeks to protect the public from, or limit exposure to, hazards that may be harmful to health. More specifically, it is concerned with the prevention, investigation, and control of infectious diseases as well as environmental hazards.	1. Actions of environmental hazard avoidance concerned with the prevention, investigation and control of hazards	1. Needs of public health 2. Knowledge and awareness	2. Enhancing skills for nurses and public health practitioners
[34]	Health protection is the protection of individuals, groups, and populations through expert advice and effective collaboration to identify, prevent, and mitigate the impacts of infectious disease, and environmental, chemical, and radiological threats.	1. Actions of protection through compliance with safety guidance 2. Actions of environmental hazard avoidance 3. Actions of harmful substance avoidance 4. Concern for social environment	1. Perception of risk of infectious disease, and environmental, chemical, and radiological threats 2. Knowledge 3. Awareness	1. Occupational health 2. Reduced healthcare costs 3. Enhanced well‐being
[35]	Health protection refers to activities which aim to protect individuals, communities and populations from both acute and chronic hazards such as infectious disease incidents and outbreaks and from environmental hazards such as chemicals, poisons and radiation.	1. Actions of environmental hazard avoidance 2. Actions of harmful substance avoidance	1. Perception of acute and chronic hazards 2. Awareness of hazards 3. Knowledge of protection and detection	1. Safety and health 2. Reduced healthcare costs 3. Enhancing environmental safety
[36]	Health protection refers to actions primarily involving the use of legal, regulatory, or enforcement mechanisms to safeguard public health.	1. Actions to safeguard public health with compliance to safety guideline 2. Concern for social environment	1. Hazards to health 2. Laws and regulations requirement	1. Safety situation 2. Increased productivity
[37]	Health protection refers to preventing potential health risks and diseases.	1. Actions of preventive healthcare 2. Personal health practice	1. Perception of potential health risks and diseases 2. Knowledge and awareness 3. Access to healthcare services	1. Available environment 2. Positive health status 3. Quality of life 4. Reduced healthcare costs
*Education (n = 1)*				
[39]	Health protection refers to actions with policies and regulatory procedures, such as legislation and regulations, and standards which protect people from hazards to health.	1. Actions of compliance with safety guidelines and requirements to protect humans	1. Perception of risk of hazards to health 2. Laws and regulations requirement 3. Access to services	1. Environmental change 2. Behavioral change
*Sociology (n = 2)*				
[40]	Health protection refers to the legal and fiscal measures which aim to prevent ill health and promote positive health status, for example the taxation of alcohol and tobacco, seat belt legislation and health and safety legislation.	1. Measures of compliance with safety guidelines and requirements to prevent illness and promote health 2. Personal health practice 3. Concern for social environment	1. Awareness of risk of ill health 2. Knowledge 3. Resources available	1. Positive health status 2. Enhanced public trust 3. Physical health
[41]	“Health protection” refers to factors in human settlements that can protect the system and the health of residents.	1. Factors to protect systems and health	1. Human needs 2. Knowledge and awareness	1. Health satisfaction 2. Increased productivity
*Management (n = 2)*				
[42]	Health protection refers to the procedures governing the occupational and environmental safety guidelines.	1. Procedures of compliance with safety guidelines	1. Perception of potential health risk 2. Knowledge 3. Resources available	1. Occupational and environmental safety
[43]	Health protection refers to all activities that directly or indirectly affect the prevention, maintenance, and improvement of the state of health of the population.	1. Activities directly or indirectly positively affecting health	1. Incidence of ill health and illness	1. People's quality of life

#### 3.2.1 Behavior of Proactive Assessment and Control to Environmental Hazards and Harmful Substances

This attribute of HPB involves proactively assessing and managing potential environmental hazards and harmful substances that could pose risks to health [26, 27, 32]. This includes behaviors such as proactively identifying and understanding potential hazards in the environment [30, 35], taking measures to minimize exposure to harmful substances (e.g., proper ventilation) [33], and implementing strategies to control or mitigate risks (e.g., proper disposal of hazardous materials) [38].

#### 3.2.2 Behavior of Passive Compliance With Safety Guidance

This attribute refers to passively following safety guidelines and regulations to protect one's health and well‐being. It involves adhering to recommended safety practices, rules, and guidelines set forth by relevant authorities or organizations [28, 36]. Examples include following workplace safety protocols [39], using protective equipment (e.g., seatbelts, helmets), and following safety instructions for handling equipment or engaging in specific activities [34, 40, 42].

#### 3.2.3 Behavior Concerned With the Social Environment

This attribute highlights the importance of considering the social environment and its impact on health. It involves behaviors that promote a healthy social environment, such as fostering positive relationships, engaging in supportive and respectful communication, and actively participating in community activities [34, 36]. Additionally, it includes behaviors that contribute to the well‐being of others, such as promoting inclusivity, supporting social justice, and advocating for a safe and healthy community [28, 40].

#### 3.2.4 Behavior for Personal Health Practice

This attribute encompasses individual actions and behaviors that contribute to personal health and well‐being [37]. It involves adopting and maintaining healthy habits, such as engaging in regular physical activity, practicing good hygiene (e.g., handwashing), getting sufficient sleep, managing stress, and maintaining a balanced diet [29].

#### 3.2.5 Behavior for Maintenance of Health

This attribute emphasizes the ongoing efforts to sustain and promote overall health. It involves behaviors that support the maintenance of good health, such as engaging in preventive measures (e.g., vaccinations, cancer screenings) [37], managing risk factors (e.g., maintaining a healthy weight, avoiding smoking) [41], and staying informed about health‐related issues [14]. Additionally, it includes behaviors that contribute to long‐term health and well‐being, such as engaging in lifelong learning, adopting positive coping strategies, and prioritizing self‐care and body checkups [29, 31, 43]. The frequency of attributes existing in the definitions of the selected articles is listed in Table 2.

**Table 2:** The frequency of attributes existing in the selected articles.

Attributes (original, from the literature)	Frequency	Attributes (final)
☑ Actions of environmental hazard avoidance concerned with the prevention, investigation, and control of hazards	1	1. Behavior of proactive assessment and control of environmental hazards and harmful substances
☑ Actions of environmental hazard avoidance	3	
☑ Activities of actively protecting or detecting health‐related factors	1	
☑ Interventions by modifying the environment	1	
☑ Actions of harmful substance avoidance	4	
☑ Measures of harmful substance avoidance to safeguard health	1	
☑ Actions of compliance with safety guidelines and requirements to protect humans	1	2. Behavior of passive compliance with safety guidance
☑ Actions of prevention and promotion by compliance with safety guidance	1	
☑ Actions of protection through compliance with safety guidance	1	
☑ Measures of compliance with safety guidelines and requirements to prevent illness and promote health	1	
☑ Procedures of compliance with safety guidelines	1	
☑ Concern with social environment	4	3. Behavior concerned with social environment
☑ Personal health practice	3	4. Behavior of personal health practice
☑ Actions of preventive healthcare	1	5. Behavior of maintenance of health
☑ Active behavior	1	
☑ Activities directly or indirectly affecting health positively	1	
☑ Behavior decreased probability of becoming ill	1	
☑ Behavior decreased probability of becoming ill	1	
☑ Factors to protect systems and health	1	

*Note:* The attributes of health protection were categorized by thematic analysis.

### 3.3 Identifying a Model Case

A model case is a pure “classic” example of the analysis concept that contains absolutely all attributes and no others [25].

In this case, we have an individual named Sarah (35 years old), working in a Cath Lab with an occupational radiation exposure problem, who exhibits all the attributes of HPB. She proactively identifies and addresses radiation distribution by radiation dosimeters and controls the hazards by creating a farther distance from the radiation source. Sarah passively adheres to safety guidance provided by health authorities and actively promotes adherence to these guidelines among her peers. She actively engages in behaviors that address social determinants of health, such as keeping better relationships with her colleagues and informing the doctor when she is going to be near a high radiation area. Sarah also practices personal health hygiene, including proper handwashing, regular exercise, and a healthy diet. Furthermore, she actively maintains her health through regular medical check‐ups, vaccination, and a balanced diet. Sarah serves as a role model for others in terms of HPB.

### 3.4 Developing Additional Cases

#### 3.4.1 Borderline Case

A borderline case is one that will be similar to the concept but different in some ways. It always contains many of the defining characteristics but may lack one or have an extra one [25].

In this case, we have an individual named Alex (40 years old), working as a worker with noise hazards, who exhibits mixed behaviors related to HPB. Alex occasionally proactively assesses noise hazards by noise detector and actively avoids the noise by moving farther away. He received training on the proper use of hearing protection devices and is required to wear them in noisy work areas. His company has isolated noisy machinery and equipment from workspaces as well as stablished quiet zones for employees to take breaks. He has a healthy diet, doing regular exercise to enhance his resilience to noise. However, he does not participate in any body examinations. This borderline case highlights the ambiguity surrounding Alex's HPB.

#### 3.4.2 Related Case

Related cases are concepts that are related to the concept of interest but are not the concept itself. Related concepts are connected or occur within a similar cluster of concepts but are not the concept [25].

Health promotion case: Kate works as an officer with musculoskeletal problems. She starts her day with morning exercise, follows a nutritious diet, practices mindfulness to manage stress, and advocates for hydration and work–life balance. Kate participates in health screenings, encourages her colleagues to prioritize well‐being, and spreads positivity and motivation throughout the workplace. Her dedication fosters a healthier and more productive environment, inspiring others to take charge of their health and well‐being. Kate's commitment to health promotion serves as an inspiration to others, encouraging them to prioritize their health and make positive lifestyle choices.

#### 3.4.3 Contrary Case

A contrary case is a clear case of “not the concept.” It is often a negative example of the concept [25].

In this case, we have an individual named John, working in a chemical factory, who lacks HPB. John neglects assessing and controlling environmental hazards and harmful substances, exposing himself to potential risks. He disregards safety guidance and shows noncompliance with recommended safety measures, putting himself and others at risk. John pays minimal attention to the social environment and does not actively engage in activities that promote the well‐being of his community. He demonstrates poor personal health practices, such as neglecting personal hygiene and engaging in unhealthy behaviors like smoking and excessive alcohol consumption. John does not prioritize maintaining his health and neglects regular medical check‐ups or preventive measures. He serves as a clear example of the lack of HPB.

### 3.5 Identifying Antecedents and Consequences

Antecedents occur before the concept of interest and are often crucial to its occurrence or required for its existence. Consequences occur because of the concept; they are the effects of the concept or the outcomes of the concept. Antecedents and consequences are often found in the original list of defining characteristics [25].

#### 3.5.1 Antecedents

The antecedents of HPB can be various workplace and environmental hazards such as physical, chemical, biological, ergonomic, or psychosocial risks [18, 45]. These hazards can arise from a variety of sources including work processes, work environment, work equipment, substances used, or human behavior. According to the literature review, they can be found in multiple disciplines, and most of these disciplines are related to humans. Antecedents can be categorized into ten aspects, as shown in Figure 2.

##### 3.5.1.1 Human Needs

Human needs refer to the fundamental requirements necessary for an individual's well‐being, such as physiological needs, safety needs, and social needs [38]. When individuals perceive threats to their health or safety, their inherent need for self‐preservation and well‐being can motivate them to engage in HPB [41, 43].

##### 3.5.1.2 Access to Resources

Access to resources encompasses the availability and affordability of the necessary tools, facilities, information, and services to engage in HPB [37, 39, 40]. Adequate resources, such as healthcare services, personal protective equipment, and educational materials, can facilitate individuals in taking actions to protect their health [26, 27, 34, 42].

##### 3.5.1.3 Awareness of Hazards and Risks

Awareness of hazards and risks refers to individuals' knowledge and understanding of potential dangers and threats to their health [14, 26, 27, 34, 35, 39, 42]. When individuals are aware of specific risks, such as exposure to harmful substances or infectious diseases, they are more likely to engage in behaviors aimed at preventing or minimizing them [28, 31, 32, 35, 37, 38, 40, 41].

##### 3.5.1.4 Knowledge

Knowledge encompasses the information and understanding individuals possess about health‐related topics, including the causes, consequences, and preventive measures related to certain health conditions or risks [37, 40, 42]. Knowledge empowers individuals to make informed decisions and engage in HPB [26, 27, 29, 30, 31, 32, 34, 35, 38, 41].

##### 3.5.1.5 Motivation

Motivation refers to the internal or external factors that drive individuals to act in a particular way. In the context of HPB, motivation can be influenced by various factors such as personal beliefs, perceived benefits and barriers, social norms, and the desire to maintain well‐being or protect loved ones [26, 28, 31].

##### 3.5.1.6 Self‐Efficacy

Self‐efficacy refers to an individual's belief in their own ability to successfully perform a specific behavior [14, 27]. When individuals have confidence in their capabilities to adopt and sustain HPB, they are more likely to overcome challenges and persist in their efforts [29].

##### 3.5.1.7 Social Support

Social support involves the assistance, encouragement, and empathy provided by individuals' social networks, such as family, friends, and communities. Positive social support can enhance individuals' motivation and provide practical assistance, making it easier for them to engage in HPB [29].

##### 3.5.1.8 Stakeholder Involvement

Stakeholder involvement refers to the active participation and collaboration of various individuals and organizations with an interest or influence in promoting HPB [30]. This can include healthcare providers, government agencies, nonprofit organizations, and community leaders working together to create supportive environments and implement effective interventions.

##### 3.5.1.9 Laws and Regulations Requirement

Laws and regulations play a crucial role in shaping individuals' behavior by establishing standards, guidelines, and legal requirements related to health protection [33, 36]. When laws and regulations require certain HPB, individuals may be more likely to comply due to the potential consequences of noncompliance [39].

##### 3.5.1.10 Safety Climate

Safety climate refers to the organizational or environmental conditions and perceptions that influence individuals' beliefs, attitudes, and behaviors related to safety. A positive safety climate, characterized by a supportive and proactive approach to health and safety, can foster a culture where HPB is valued and prioritized [31].

#### 3.5.2 Consequences

The consequences of these hazards can range from minor injuries or illnesses to severe or even fatal consequences. These consequences can have a significant impact on the affected worker, their family, and the wider community. Consequences can also result in significant costs to the employer, such as increased absenteeism, decreased productivity, and legal or compensation costs. The consequences of HPB can be found in multiple disciplines, according to the literature review. They can be categorized into six aspects, as shown in Figure 2.

##### 3.5.2.1 Enhanced Public Trust

When individuals engage in HPB, it can lead to enhanced public trust [28, 31, 32, 40]. By actively taking steps to protect their health and well‐being, individuals demonstrate a sense of responsibility and care, which can foster trust among the public. This trust can extend to healthcare providers, institutions, and authorities involved in promoting health and safety [33].

##### 3.5.2.2 Enhanced Well‐Being

Engaging in HPB contributes to enhanced well‐being [14, 27, 34, 41]. By adopting preventive measures and practicing healthy behaviors, individuals can reduce the risk of illness, injury, or other adverse health outcomes. This, in turn, can lead to improved physical, mental, and emotional well‐being, allowing individuals to lead healthier and more fulfilling lives.

##### 3.5.2.3 Safety Situation

HPB positively impacts the safety situation. By taking appropriate precautions and following safety guidelines, individuals can minimize the occurrence of accidents, injuries, or incidents that may compromise their health or safety [26, 27, 28, 33, 35, 36, 37, 39, 42]. This contributes to creating safer environments for individuals and communities.

##### 3.5.2.4 Quality of Life

Engaging in HPB can improve quality of life. By prioritizing health and taking proactive steps to prevent illness or injury, individuals can maintain or improve their overall health status [26, 27, 29, 30, 32, 34, 37, 40, 43]. This can result in a higher quality of life, including increased productivity, greater participation in daily activities, and improved physical and mental functioning.

##### 3.5.2.5 Cost Savings

HPB can lead to cost savings. By preventing or minimizing health risks, individuals can reduce healthcare costs associated with treating illnesses or managing chronic conditions. Additionally, preventive measures and healthy behaviors can help individuals avoid costly medical interventions or hospitalizations [31, 34, 35, 37]. This, in turn, can contribute to overall cost savings for individuals, healthcare systems, and society as a whole.

##### 3.5.2.6 Sustainability

Engaging in HPB promotes sustainability. By adopting behaviors that prioritize health and well‐being, individuals contribute to sustainable practices and systems [29, 30, 36, 38, 40, 41]. For example, preventive healthcare practices can lead to a more efficient use of healthcare resources, reduced burden on healthcare systems, and a focus on long‐term health outcomes. This sustainable approach supports the well‐being of individuals, communities, and future generations.

### 3.6 Defining Empirical Referents

Empirical referents are the means by which one can determine if a concept exists in any given circumstances, and they can always be operational measures of the defining characteristics of the concept [25].

Based on the five attributes of health protection, the defined empirical referents could be as follows:

#### 3.6.1 Behavior of Proactive Assessment and Control of Environmental Hazards and Harmful Substances

These involve observational assessments of individuals' adherence to safety protocols and use of protective equipment in hazardous environments. Self‐report questionnaires or interviews can be used to measure individuals' knowledge of environmental hazards and their practices for assessing and controlling them while environmental monitoring techniques can measure exposure levels to harmful substances in the environment.

#### 3.6.2 Behavior of Passive Compliance With Safety Guidance

Compliance checklists or rating scales can be used to assess individuals' adherence to specific safety guidelines or protocols. Self‐report surveys or interviews measure individuals' self‐reported compliance with safety practices and their understanding of safety guidance. Observation or video monitoring of individuals' behaviors in real‐world or simulated situations can be used to evaluate their compliance with safety guidance.

#### 3.6.3 Behavior Concerned With the Social Environment

Surveys or questionnaires assess individuals' engagement in community health initiatives or involvement in social support networks. Scales measure individuals' attitudes and beliefs about social determinants of health and their commitment to addressing them, and document analysis of individuals' participation in advocacy or community health‐related activities can also be performed.

#### 3.6.4 Behavior of Personal Health Practice

These can involve self‐report assessments of individuals' engagement in specific health behaviors, such as physical activity, hygiene practices, dietary habits, stress management, and tobacco or substance use. Objective measurements like pedometers or activity trackers can be used to quantify individuals' physical activity levels while biological markers or laboratory tests evaluate individuals' adherence to medical treatment plans or their overall health status.

#### 3.6.5 Behavior of Maintenance of Health

Medical records or claims data can be used to track individuals' adherence to regular check‐ups, screenings, and vaccinations. Self‐report measures assess individuals' engagement in self‐care activities, health monitoring, and health promotion behaviors, and health‐related quality of life assessments measure individuals' subjective well‐being and perceived maintenance of health.

### 3.7 Conceptualization of Health Protective Behavior in Occupational Health Practice

Consequently, HPB can be defined as “action involving actively assessing and controlling environmental hazards and harmful substances, complying with safety guidance, demonstrating concern for the social environment, practicing personal health habits, and maintaining overall health.”

## 4 Discussion

The concept of HPB was redefined through the process of concept analysis by Walker and Avant. HPB in occupational health practice will guide workers in seeking health and safety related to work which can be influenced by the antecedents, and it will provide a process for decision‐making and action by the encouraging consequences. HPB has been tested by four competing theories, including the health belief model (HBM), protection motivation theory (PMT), subjective expected utility theory (SEU), and the theory of reasoned action (TRA) [45]. Various factors influencing HPB have been identified, such as psychological, demographic, and workplace factors. Ping et al. [46] have developed an instrument to measure the community group by different dimensions.

However, the inconsistencies that have occurred in the intercorrelations among HPBs and in its underlying dimensions may suggest an inherent instability in the concept of HPB [47]. The identified inconsistencies in intercorrelations among HPBs likely stem from three key factors: contextual variability, measurement heterogeneity, and diverse theoretical frameworks. Studies focusing on healthcare workers often emphasize compliance with infection control protocols, while construction industry research prioritizes hazard assessment and equipment safety [18]. Such sector‐specific risks lead to divergent behavioral priorities, complicating cross‐study comparisons. Additionally, methodological disparities, such as reliance on self‐reports versus observational data, introduce measurement biases [47]. To resolve these issues, future research should standardize HPB measurement tools, adopt context‐sensitive frameworks that account for industry‐specific hazards and organizational cultures, integrate mixed‐methods approaches to capture both subjective perceptions and objective behaviors. By addressing these gaps, the reliability and applicability of HPB as a unified concept can be strengthened.

The redefined HPB framework offers actionable strategies for enhancing workplace safety. Workplace policies should include mandatory safety audits and empower employees to report risks. This approach allows for proactive hazard control using digital reporting platforms. Ensuring compliance with safety protocols through real‐time monitoring will be more effective [48]. To support this framework, we should cultivate a “safety‐first” culture within the organization through team‐based incentives and leadership training [49]. Safety training programs should include modular training focusing on each HPB attribute. This can consist of hazard identification workshops, scenario‐based drills for emergencies like fire evacuations, and communication skills training to manage psychosocial risks such as conflict resolution.

Based on the recent studies, the phenomenon of “attitude‐practice separation” exists in HPB. Most researchers suggest testing more theories of paradigms to find the possible moderating variables between attitude and behavior. Therefore, the research needs to incorporate more perspectives, such as different work conditions and target population testing, theoretical and empirical evidence testing, factor exploring and interaction effects of factors, and instrument development for creating a series of interventions to enhance HPB in occupational health practice.

## 5 Limitations

This study has some limitations regarding the process of concept analysis which should be disclosed. (1) This study only focuses on HPB in the field of occupational health practice related to health keeping in a worker's career. Even though Walker and Avant [25] suggest thinking in a generalizable way but only focusing on a specific field, some research has revealed the meaning changes in different realms, so specification will be useful for research [50]. (2) Only one method of concept analysis was utilized; different methods may lead to different results.

## 6 Conclusion

This analysis proposes a new conceptualization of HPB in occupational health practice. Nineteen articles related to HPB in occupational health practice were selected as the materials for analysis. Five attributes, ten antecedents, and six consequences were identified. Empirical referents, model case, borderline case, related case, and contrary case were found, and HPB was redefined. A clearer concept of HPB as well as the analyzed and redefined components of HPB in occupational health practice will benefit occupational health and nursing practice. Furthermore, the analysis of HPB can be used for exploring the influencing factors, instrument development, and intervention development for workers.

## Author Contributions

**Fenggang Liu:** methodology, conceptualization, formal analysis, funding acquisition, writing – original draft. **Juanjuan Wang:** methodology, conceptualization, formal analysis, writing – original draft. **Weeraporn Suthakorn:** methodology, conceptualization, formal analysis, supervision, writing – original draft. **Li Liao:** methodology, conceptualization, formal analysis, supervision, writing – original draft. All authors have read and approved the final version of the manuscript.

## Ethics Statement

Ethical approval of the study was granted from the First Affiliated Hospital of University of South China, No: 2024LL0722001.

## Conflicts of Interest

The authors declare no conflicts of interest.

## Transparency Statement

The lead author Fenggang Liu affirms that this manuscript is an honest, accurate, and transparent account of the study being reported; that no important aspects of the study have been omitted; and that any discrepancies from the study as planned (and, if relevant, registered) have been explained.

## Data Availability

The data that support the findings of this study are available on request from the corresponding author. The data are not publicly available due to privacy or ethical restrictions. Weeraporn Suthakorn had full access to all of the data in this study and took complete responsibility for the integrity of the data and the accuracy of the data analysis.
